# Supplementary material for: Characterization of Biogenic PbS Quantum Dots
Source: Int J Mol Sci. 2023 Sep 15;24(18):14149. doi: 10.3390/ijms241814149 (PMC10531774; doi:10.3390/ijms241814149)
Supplement: Supplementary file 1 [file ijms-24-14149-s001.zip › ijms-2539968-supplementary.pdf]

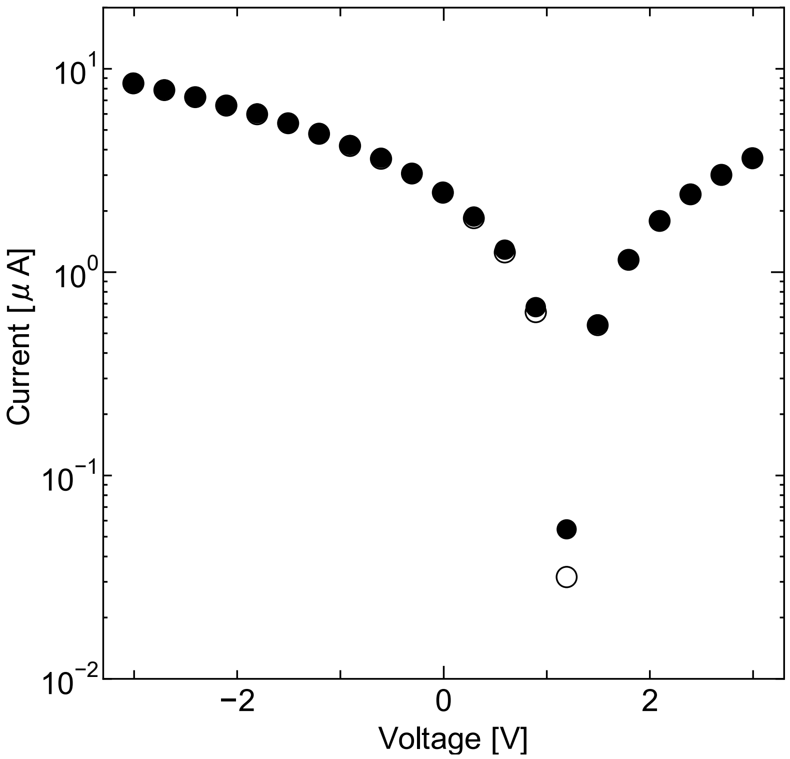

Supplemental Figure S1. Current–voltage measurements of extracted PbS from the cells grown with 5mM lead acetate. Open circle: with illumination, closed circle: without illumination.
